# Rudolph Virchow’s Public Health Engagement and Theodor Khoury’s Legacy

**DOI:** 10.31744/einstein_journal/2024ED01003

**Published:** 2024-05-09

**Authors:** Consolato M. Sergi

**Affiliations:** 1 Children’s Hospital of Eastern Ontario University of Ottawa Ottawa ON Canada Anatomic Pathology, Children’s Hospital of Eastern Ontario, University of Ottawa, Ottawa, ON, Canada.; 2 Department of Laboratory Medicine and Pathology University of Alberta Hospital Edmonton Alberta Canada Department of Laboratory Medicine and Pathology, University of Alberta Hospital, Edmonton, Alberta, Canada.

Rudolph Ludwig Carl Virchow (1821-1902), the founder of Modern Pathology and Clinical Science, was a highly productive author.^(1)^ The world-famous book “Cellular Pathology” (1858) is universally considered the foundation of contemporary pathology.

## Virchow: the multitalented physician

**Figure 1:**
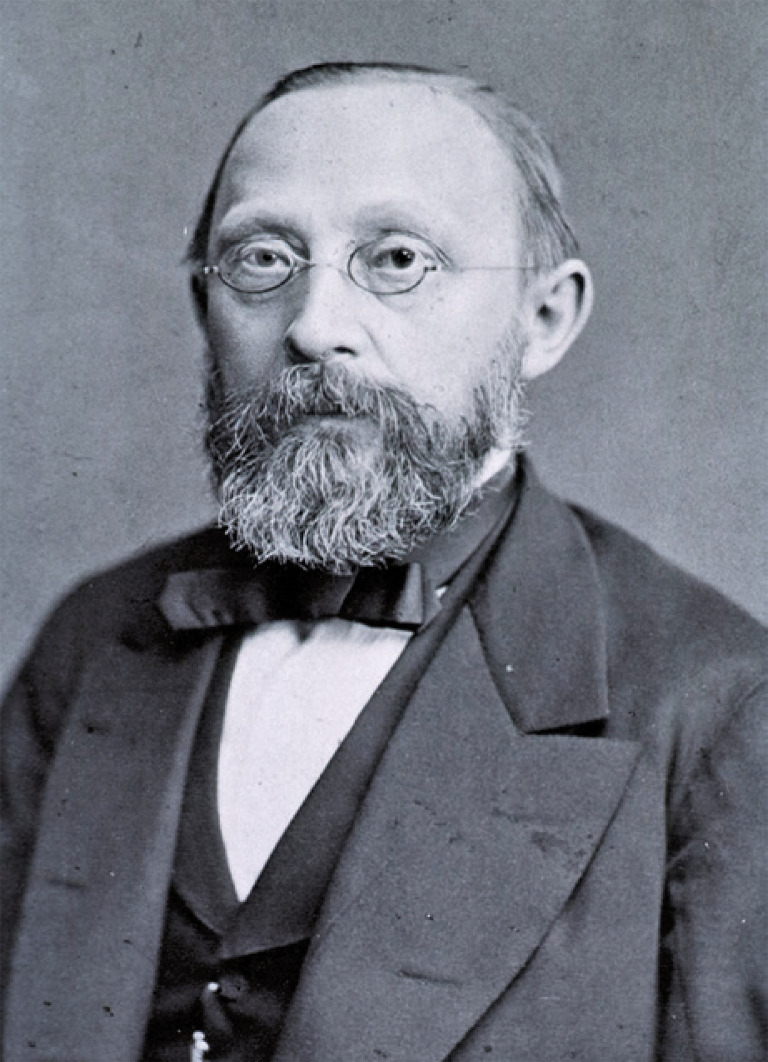
Photograph of Rudolf Virchow Source: The National Library of Medicine. Rudolph L. K. Virchow. NLM Image ID: B025666 [cited 2024 Apr 9]. Available from: https://collections.nlm.nih.gov/catalog/nlm:nlmuid-101431205-img

Virchow was a multifaceted German physician (Figure 1). He excelled in anthropology, biology, pathology, and politics. Virchow pursued his medical education at Friedrich-Wilhelms-Universität in Berlin. While at the Charité hospital, he studied the *typhus* outbreak in Upper Silesia (1848), establishing the basis for public health in Germany.^(2)^ Virchow linked the *Rickettsia prowazekii* outbreak to poverty.^(2,3)^ Virchow contended that eradicating social disparity was the sole means to avert future epidemics.^(4)^ Virchow’s crusade against antisemitism was extraordinary, prompted by his revelation of the plight endured by the Jewish population, who had been subjected to enslavement, marginalization, and ridicule by various nations throughout history. Virchow made public declarations for the elimination of racial animosity and religious extremism reminiscent of medieval times.

## Theodor Khoury’s Legacy

**Figure 2:**
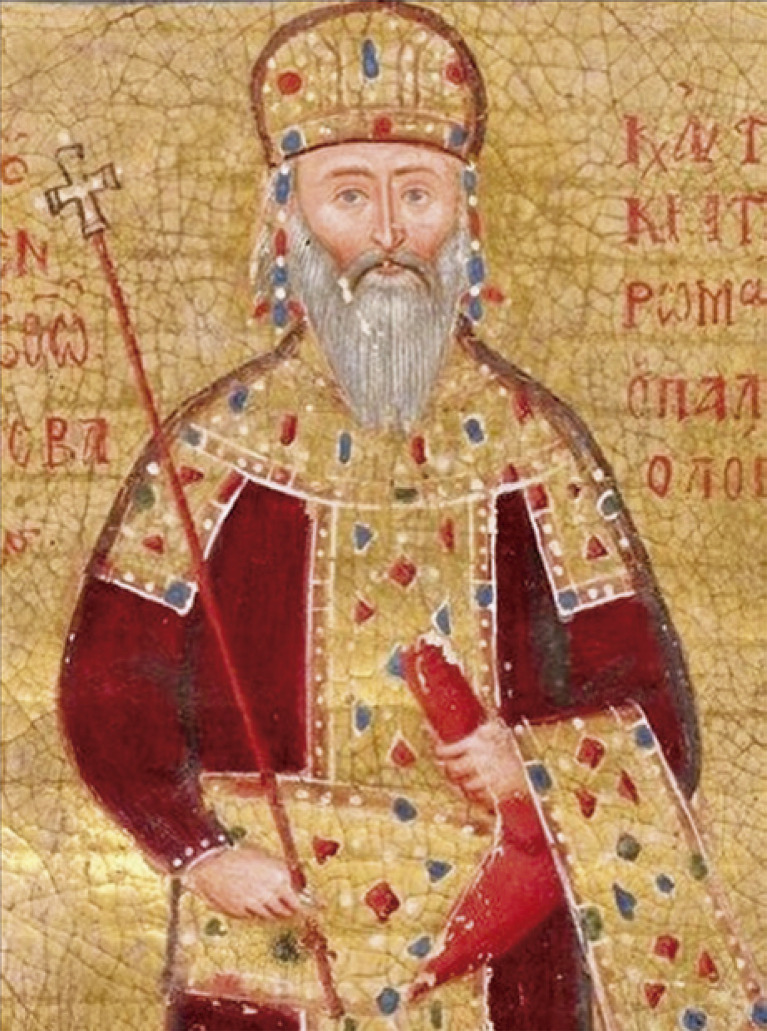
Manuel II Palaiologos Source: Wikimedia Foundation, Inc. Manuel II Palaiologos. [cited 2024 Apr 9]. Available from: https://en.wikipedia.org/wiki/Manuel_II_Palaiologos

2023 will probably also be recalled for the death of Adel Theodor Khoury, a Lebanese theologian who studied philosophy and Orientology in Beirut. Later, he obtained a doctoral degree in Lyon, France, and held from 1970 until 1993, the Chair of General Religious Sciences (*Allgemeine Religionswissenschaft*) in the Westfälische Wilhelms-Universität Münster, Germany. Khoury is distinguished for his publications and efforts toward interfaith understanding and dialogues promoting fervid communication platforms.^(5)^ The dialogue of Manuel II Palaiologos (Figure 2), a Byzantine emperor, with an “educated Persian” received wide attention, scrutiny, and misunderstanding in 2006 during a magisterial lecture of the late Pope Benedict XVI in Germany.^(6)^ This dialogue, which occurred around 1391 CE in Ankara, examined Christianity and Islam. In the debate (διάλεξις), the emperor discusses the topic of the “holy” war. The emperor presumably possessed knowledge of verse 2, 256 from the Surah that “there is no coercion in matters of faith,” arguing that holy wars are unacceptable.^(7)^ The lecture was blatantly misunderstood, arousing indignation. However, the Byzantine and subsequent Christian leaders missed the remarkable and crucial role of Muslims in the steps of Western civilization. The excellence in astronomy, mathematics, natural sciences, and literature brought by the Islamic Golden Age into Western civilization is immense. This era, which is conventionally dated from the 8^th^ century to the 13^th^ century CE, is universally considered a period of cultural blossoming and respectful tolerance in the history of Islam. During this prosperous age, the Muslims exhibited a genuine and convincing interest in integrating the scientific knowledge of the countries and civilizations that had been seized.^(8)^ Muslim heritage is underscored, scorned, and depraved by Hamas and other terroristic organizations. It is heartbreaking that very few Muslim communities condemned the October 7 massacre. The evil acts perpetrated by terrorists must not obfuscate the positive achievements of the Islamic culture. Emphasizing and declaring the beauty and the formidable steps reached through this culture in our civilization is critical nowadays, but firm condemnation of evil acts is also necessary.

## Medical professionals’ reaction

The most recent weeks have been characterized by a substantial number of unpleasant and near-lethal incidents involving Jewish students and fellows in academic institutions in Europe and North America.^(9)^ These individuals have been ostracized, marginalized, and scorned. The actions perpetrated by Hamas on Israeli victims were exceedingly abhorrent. Consequently, the last recourse for anti-Israel extremists to sway public opinion is to outright deny the occurrence of such events. Individuals who aim to refute the occurrence of the Holocaust tragically employ a similar strategy. Eugen Kogon (1903–1987), a German historian and Nazi concentration camp survivor, wrote a detailed book on Nazi crimes. Recently, most medical practitioners and academics have refrained from making public pronouncements or remarks regarding the Simchat Torah massacre. However, some organizations have documented numerous medical professionals whose words or actions concerning current events unmistakably exhibit unbridled hatred against Jews.^(9)^ Kingsbury and Greene identified 42 groups, of which 31 (74%) had issued an official communication regarding Ukraine, whereas just 11 (26%) had done the same for Israel. Medical associations were nearly three times more inclined to issue statements regarding the Ukraine crisis than those in Israel. However, there were discernible disparities in the responses of the 11 medical societies that commented on Ukraine and Israel. Nearly 75% of medical groups expressed the necessity to address the conflict in Ukraine, whereas roughly 75% remained silent regarding the war in Israel. It may appear perplexing that Jews, a collective that has endured the immense anguish of the Holocaust, along with numerous pogroms, social marginalization, and psychological distress, can be easily portrayed as oppressors.^(9)^

German pathologist and statesman Rudolf Virchow and German historian Theodor Khoury were brave men.^(10)^ The Middle East tragedy and the post-event commentaries and vilifications are bound to a status of nihilism and relativism, which afflict our world and our culture. Western civilization is not innocent, and numerous complicities of Western culture and Christianity have been associated with the killing of heretics, Jews, and First Nations. In 2023, we recalled Virchow’s 175^th^ anniversary of his report on Upper Silesia’s *typhus* triggering his public health and politics engagement and reported on the death of Khoury.^(11)^ It is imperative to support and emphasize that interfaith dialogues are still critical for peace and prosperity in our world. Fighting poverty, poor education, pseudo-indoctrination, and brainwashing is crucial for the future of medicine and our society.
